# Modeling acoustic cavitation with inhomogeneous polydisperse bubble population on a large scale^☆^

**DOI:** 10.1016/j.ultsonch.2022.106060

**Published:** 2022-06-09

**Authors:** Sergey Lesnik, Atiyeh Aghelmaleki, Robert Mettin, Gunther Brenner

**Affiliations:** a Clausthal University of Technology, Institute of Applied Mechanics, Adolph-Roemer-Straße 2A, 38678 Clausthal-Zellerfeld, Germany; b University of Göttingen, Third Institute of Physics, Friedrich-Hund-Platz 1, 37077 Göttingen, Germany

**Keywords:** Acoustic cavitation modeling, Ultrasonic reactors, Bubble dynamics, Bubble population, OpenFOAM

## Abstract

A model for acoustic cavitation flows able to depict large geometries and time scales is proposed. It is based on the Euler–Lagrange approach incorporating a novel Helmholtz solver with a non-linear acoustic attenuation model. The method is able to depict a polydisperse bubble population, which may vary locally. The model is verified and analyzed in a setup with a large sonotrode. Influences of the initial void fraction and the population type are studied. The results show that the velocity is strongly influenced by these parameters. Furthermore, the largest bubbles determine the highest pressure amplitude reached in the domain, which corresponds to the Blake threshold of these bubbles. Additionally, a validation is performed with a small sonotrode. The model reproduces most of the experimentally observed phenomena. In the experiments, neighboring bubbles are found which move in different directions depending on their size. The numerical results show that the responsible mechanism here is the reversal of the primary Bjerknes force at a certain pressure amplitude.

## Highlights

• A new model for acoustic cavitation flows was developed, implemented, verified and validated.
• The model allows new insights into the behavior of cavitation flows.
• The bubble and flow velocities are strongly influenced by the population type and the void fraction.
• The largest bubbles determine the highest pressure amplitude reached in the domain, which corresponds to the Blake threshold of these bubbles.

## Graphical abstract

**Figure:**
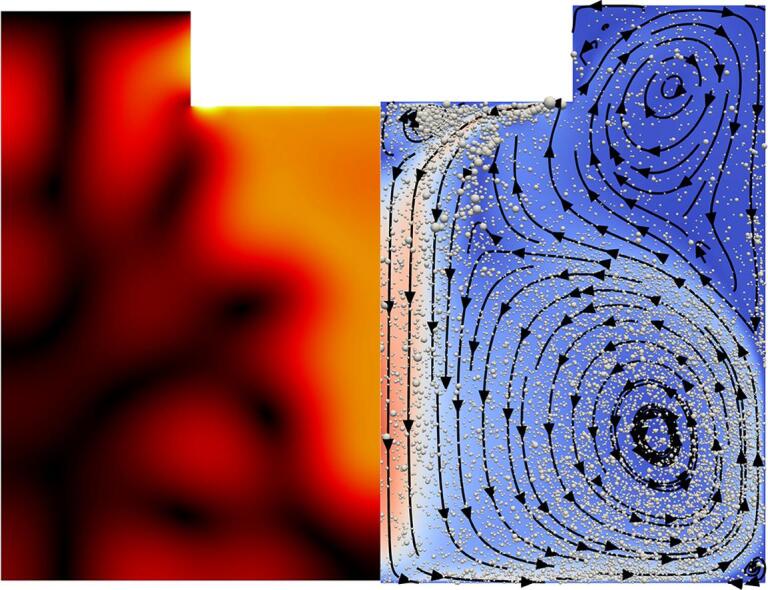

## 1 Introduction

The numerical methods describing cavitating flows differ in the treatment of the liquid (also called carrier) and of the vapor/gas (also called dispersed) phases. The available approaches may be divided into five categories:

• Single Euler phase
• Lagrangian tracking
• Euler-Euler
• Euler–Lagrange
• Combined Euler-Euler/Euler–Lagrange

Single Euler phase approaches describe the acoustic pressure field and therefore consider only the liquid phase. In case of acoustic cavitation, the acoustic pressure field is the field being discretized and solved. The bubbles do not directly participate in the modeling procedure but their influence is included through algebraic equations. The flow motion is usually not considered. Dähnke and Keil [12], [13] developed a Finite Difference code with a single Euler phase representing the liquid. They introduced the influence of bubbles using the linear damping model of Commander and Prosperetti [10], whereby the bubbles are assumed to oscillate linearly during the cavitation process. The recent experiments of Žnidarčič et al. [56] with the comparable setup demonstrated that the maximum acoustic pressures are an order of magnitude lower than predicted by Dähnke et al. [13]. Louisnard [29] showed that realistic acoustic pressure amplitudes can be depicted by an Eulerian model, when the non-linear bubble oscillations and the resulting high damping of the acoustic field are considered. The non-linear damping model [28] is a part of the present work and will be described in detail later.

Lagrangian tracking methods focus on the calculation of the motion of cavitation bubbles. The latter are introduced as discrete particles, whereby each particle is tracked individually in space and time. The acoustic pressure field is usually provided as an initial field, which stays stationary during the calculation. This is also called one-way coupling, because only the bubbles are influenced by the sound field and not vice versa. First attempts to describe motion of the acoustic cavitation bubbles by Lagrangian tracking date back to works of Mettin et al. [36], [35], who depicted the bubble motion as Lagrangian particles. A stationary pressure field was provided as an input, which acted on the bubbles as a momentum source. Flow field and bubble interactions with both fields and with each other were neglected. In a recent work, Sugita [50] used the Lagrangian particles approach within the frame of the Boundary Element Method (BEM) to study the behavior of cavitation bubble clouds. The main focus was on the interaction of the bubbles with each other and with the reactor walls.

The Euler-Euler method depicts both the gaseous (cavitation bubbles) and the liquid phase as continua. Both phases influence each other, which is also known as two-way coupling. Mottyll and Skoda [37] used a solver with a barotropic cavitation model, which is able to depict the liquid–vapor mixture as two Eulerian phases. The studied setup consisted of sonotrode immersed into water, whereby the sonotrode was driven with a high amplitude. This resulted in a dense bubble formation, which evolved into a cloud cavitation, where only few oscillating bubbles were present and the majority of the vapor formed a large oscillating non-spherical volume beneath the sonotrode. The Euler-Euler approach is often used in terms of the hydrodynamic cavitation, e.g. Blume and Skoda [5] utilized the same solver as in [37] and investigated cloud cavitation at a hydrofoil with a circular leading edge derived from a centrifugal pump blade. A correct identification of erosion sensitive wall zones compared to the measurements was reported. Another example of a study of cavitation at a hydrofoil is the work of Zwart et al. [57].

The Euler–Lagrange method deals only with the liquid as a continuum but treats the bubbles as particles. Similar to the Euler-Euler method a two-way coupling is possible. If the bubbles are able to interact with each other as well, the coupling is called four-way. In the context of the acoustic cavitation, Fuster and Colonius [16] developed a two-way technique, which provides an outstanding feature: The oscillating bubbles at high driving pressure amplitudes emit acoustic waves. The emitted acoustic pressure is lower than the one of a sonotrode but it has still a significant impact on the neighboring bubbles. Jamshidi and Brenner [19] worked on the acoustic cavitation models applied to the process engineering and surface cleaning. The approach included the non-linear damping model [29]. The calculations were restricted to 2D geometries and a small number of Lagrangian particles.

Combined Euler-Euler/Euler–Lagrange models consider the gas phase in two frames of reference: either Eulerian or Lagrangian. The criteria to decide which frame is used at a specific location and time differ from one model to another but usually the size of the gaseous structure is used. This method is mostly applied to the hydrodynamic cavitation setups. Yakubov et al. [54] developed a two-way coupling approach where the bubble breakup was taken into account by utilizing a probabilistic model. The Euler–Lagrange method was employed only at the nucleation site of the bubbles, which was located at the propeller blade tip. A more sophisticated ansatz was chosen by Vallier [52] who treated the gas phase by the appropriate method depending on the size of the cavity. If it is so small that a spherical bubble forms, it is represented as a Lagrangian particle. If the cavity is large, it is resolved by the Eulerian frame of reference. Similar approaches were used by Lidtke [27], Peters and ElMoctar [43], Peters [42].

The overview above demonstrates that the acoustic cavitation features a high level of physical complexity and a wide range of spatial and temporal scales. The latter is the reason for the existence of numerous approaches to model cavitation. Depending on the application, different scales play major role and the numerical methods fitting best the needs of the study should be employed. The present work focuses on an approach capable of representing cavitation flow throughout the entire geometry of the chosen setup. One of the main goals is to predict the distribution of the acoustic pressure amplitude since the cavitation intensity depends mainly on this quantity. Furthermore, for an analysis of the flow, information about the bubble locations and velocities must be available. We choose the Euler–Lagrange method since it has the best trade-off between efficiency and accuracy for the given task.

The present paper is organized as follows. In Section 2, we describe the components of the present model. Our approach utilizes several techniques such as a description of bubble radial dynamics by an ODE, the Finite Volume Method for dealing with the fluid dynamics, a treatment of acoustics in the frequency domain and dealing with the bubble motion in the Lagrangian frame. Section 3 describes how these submodels interact with each other. A particular attention is paid to the coupling of the wave propagation with the acoustic damping caused by the oscillating bubbles. Since the underlying equations are highly non-linear, we tackle this problem with an algorithm based on the Newton–Raphson method. The resulting solver is verified with [29] in the first part of Section 4. Furthermore, a 2D axisymmetric geometry with a 12 cm sonotrode is studied with the focus on the flow, acoustic pressure and bubble motion. Additionally, we quantify the influences of void fraction and excitation power on the behavior of the cavitation flow. Finally, we consider a 3D geometry with a 1 cm sonotrode in Section 5. Here, we also present experimental results to confront the findings from the calculations.

## 2 Model

### 2.1 Bubble radial dynamics

The overall model of sound field and cavitation bubble distribution is based on the dynamics of individual spherical bubbles. There exist several models that describe the dynamics of such a driven single bubble oscillation, which differ in their complexity and accuracy. We use the Keller-Miksis (KM) equation [20], which is characterized by higher accuracy and numerical stability compared to the simpler Rayleigh-Plesset equation [48]. It is also employed by Louisnard [28], whose model for sound propagation is a part of our present work and accounts for the non-linear attenuation, which will be described later. We use the KM formulation studied by Prosperetti and Lezzi [46] and add two correction terms regarding viscosity and surface tension similar to Lauterborn and Kurz [22], which are important in case of inertial cavitation:

(1) $$\begin{array}{c} \left(1-\frac{\dot{R}}{c}\right)R\ddot{R}+\left(1-\frac{\dot{R}}{3c}\right)\frac{3}{2}{\dot{R}}^{2}\\ \;=\frac{1}{\rho }\left[\left(1+\frac{\dot{R}}{c}\right)\left(p_{g}-P_{exc}sin\left(\omega t\right)-p_{\infty }\right)+\frac{R{\dot{p}}_{g}}{c}-\frac{4\mu \dot{R}}{R}-\frac{2\sigma }{R}\right], \end{array}$$

where *R* is the bubble radius, ρ,c and μ are the density, speed of sound and dynamic viscosity of the surrounding liquid, σ is the surface tension. Note that we leave the quantities describing the liquid without subscripts. The overdots denote differentiation with respect to time. Quantity $p_{g}$ is the gas pressure inside the bubble, $P_{\mathrm{exc}}$ is the (acoustic) excitation pressure amplitude at the bubble’s center in the absence of the bubble and $p_{\infty }$ is the pressure in the undisturbed liquid.

**Fig. 1:**
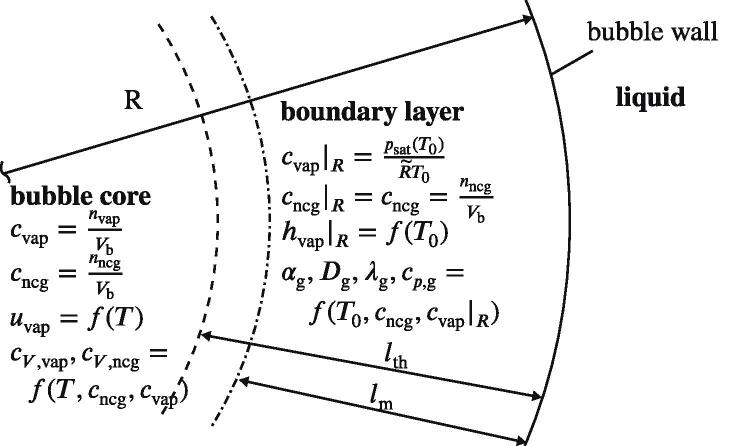
Evaluation of properties for the bubble core and wall.

Louisnard’s model [28] provides attenuation due to viscous and thermal effects and, therefore, accurate gas pressure and temperature computations are required. Toegel et al. [51] demonstrated that the gas temperature is strongly influenced by the vapor, which enters and leaves a cavitation bubble during oscillations. We use their approach and introduce two additional equations for the temperature and amount of substance of the gas mixture inside the bubble. Toegel et al. [51] split the bubble into two parts: a homogeneous bubble core and a boundary layer being in thermal equilibrium with the liquid. Fig. 1 illustrates this division. In case of the mass transport, the boundary of the splitting is at a distance of the mass diffusion length $l_{m}$ from the bubble wall. To account for the mixture, we split $n_{g}$ in a condensable vapor part $n_{\mathrm{vap}}$ and a non-condensable gas part $n_{\mathrm{ncg}}$ (air in this study). The latter is constant and the former is accounted for by a differential equation resulting from Fick’s law:

(2) $${\overset{˙}{n}}_{\mathrm{vap}}=A_{s}D_{g}\frac{c_{\mathrm{vap}}{|}_{R}-c_{\mathrm{vap}}}{l_{m}},$$

with the vapor concentration inside the bubble core $c_{\mathrm{vap}}$, the concentration at the bubble wall $c_{\mathrm{vap}}{|}_{R}$, and the bubble surface area $A_{s}$, respectively. Quantity $D_{g}$ is the diffusion coefficient of the vapor through the non-condensable gas, which is calculated by means of a molecular dynamics model [3, p. 526]. The fraction term in (2) builds an approximation for the concentration gradient at the bubble wall and utilizes $l_{m}$. Fig. 1 also describes how the quantities in the two regions are calculated, e.g. the subscript ${|}_{R}$ means that the quantity is evaluated at the distance *R* from the bubble center, namely, at the bubble wall. The concentration of the non-condensable gas is assumed to be spatially constant inside the bubble. Most of the parameters are directly or indirectly dependent on temperature, whereby *T* refers to the bubble core and $T_{0}$ to the liquid and, therefore, to the boundary layer since it is assumed to be in thermodynamic equilibrium with the surrounding as stated above. We distinguish which region the variable belongs to by indicating it as a function either of *T* or $T_{0}$ where appropriate.

The diffusion length $l_{m}$ may be estimated from the dimensional analysis resulting in $l_{m}=\sqrt{D_{g}\tau }$, where the characteristic time τ is approximated by $\frac{R}{\left|\overset{˙}{R}\right|}$. As outlined by Toegel et al. [51], $l_{m}$ in this form may exceed the bubble radius *R* and, thus, a cutoff length has to be introduced, which represents the upper bound of the mass diffusion length. They find this to be R/π by analyzing an analytical solution of the pure diffusion PDE. Preston et al. [45] utilized fully-resolved 1D calculations from which they concluded, that the correct cutoff length is R/5. We incorporate the latter correction and the final form of the diffusion length is

(3) $$l_{m}=\min\left(\sqrt{D_{g}\frac{R}{\left|\overset{˙}{R}\right|}},\frac{R}{5}\right)\text{.}$$

One last differential equation is provided for the temperature inside the bubble, which is a result of applying the second law of thermodynamics:

(4) $$\overset{˙}{T}=\frac{-p_{g}\overset{˙}{V}+\overset{˙}{Q}+{\overset{˙}{n}}_{\mathrm{vap}}(h_{\mathrm{vap}}{|}_{R}-u_{\mathrm{vap}})}{n_{\mathrm{vap}}c_{V,\mathrm{vap}}(T)+n_{\mathrm{ncg}}c_{V,\mathrm{ncg}}(T)},$$

where $h_{\mathrm{vap}},u_{\mathrm{vap}}$ and $c_{V,i}$ are respectively specific enthalpy of vapor, specific internal energy of vapor and isochoric specific heat capacities of the corresponding components. We use NASA9 coefficient polynomial parameterization [32] to compute the specific heats. The heat flux $\overset{˙}{Q}$ through the bubble surface follows from Fourier’s law and by incorporating linear interpolation for the temperature gradient:

(5) $$\overset{˙}{Q}=A_{s}\lambda_{g}\frac{T_{0}-T}{l_{\mathrm{th}}},$$

with $\lambda_{g}$ being the thermal conductivity of the gas mixture. We use a mixing rule according to Bird et al. [3, p. 276] to compute $\lambda_{g}$. A similar logic as in case of the mass balance applies to the definition of the thermal diffusion length:

(6) $$l_{\mathrm{th}}=\min\left(\sqrt{\alpha_{g}\frac{R}{\left|\overset{˙}{R}\right|}},\frac{R}{5}\right),$$

where $\alpha_{g}$ is the thermal diffusivity of the gas mixture:

(7) $$\alpha_{g}=\frac{\lambda_{g}}{\rho_{g}c_{p,g}}\text{.}$$

In case of the mixture of water vapor and air, the denominator becomes

(8) $$\rho_{g}c_{p,g}=\left(4c_{\mathrm{vap}}{|}_{R}+3.5c_{\mathrm{ncg}}\right)\overset{∼}{R}\text{.}$$

The last property needed to close the set of equations is the gas pressure $p_{g}$ from (1). In order to depict the bubble collapse correctly we use the ideal gas law with an additional covolume term $V_{\mathrm{hc}}$:

(9) $$p_{g}=\frac{n_{g}\overset{∼}{R}T}{V_{b}-V_{\mathrm{hc}}},$$

where $\overset{∼}{R},n_{g},T,V_{b}$ are the gas constant, amount of gas substance, gas temperature and volume of a bubble’s interior respectively. The covolume term comes from the Van der Waals equation, which accounts for the smallest possible volume of the molecules, so-called hard-core volume. Numerical experiments show that the covolume term is also crucial for the numerical stability. Since our model accounts for the mass transfer, $V_{\mathrm{hc}}$ is a time dependent property:

(10) $$V_{\mathrm{hc}}(t)=\frac{4}{3}\pi {\left(\frac{R_{\mathrm{eq}}(t)}{b_{\mathrm{hc}}}\right)}^{3},$$

where $R_{\mathrm{eq}}(t)$ is the equilibrium bubble radius and $b_{\mathrm{hc}}=8.86$ according to Toegel et al. [51]. Note that $R_{\mathrm{eq}}$ is also dependent on time. It is calculated by considering the gas pressure at a fictional equilibrium with the instantaneous gas amount and temperature at time $t_{*}$:

(11) $$p_{\infty }+\frac{2\sigma }{R_{\mathrm{eq}}(t_{*})}=\frac{n_{g}(t_{*})\overset{∼}{R}T(t_{*})}{\frac{4}{3}\pi R_{\mathrm{eq}}(t_{*})\left(1-b_{\mathrm{hc}}^{-3}\right)},$$

and after rearranging we arrive at a cubic equation which has only one real root:

(12) $$p_{\infty }R_{\mathrm{eq}}^{3}(t_{*})+2\sigma R_{\mathrm{eq}}^{2}(t_{*})-\frac{3n_{g}(t_{*})\overset{∼}{R}T(t_{*})}{4\pi \left(1-b_{\mathrm{hc}}^{-3}\right)}=0\text{.}$$

With the root, we are able to determine $V_{\mathrm{hc}}$.

Eqs. (1), (2), (4) build a system of ordinary differential equations. Solving this system delivers a time resolved information about the bubble radius, bubble wall velocity, temperature and amount of substance of vapor inside the bubble. How these quantities couple to the acoustic attenuation model is shown in section 2.3.

### 2.2 Blake threshold

The Blake threshold pressure was proposed by Blake [4]. If the excitation of a bubble is strong enough, the bubble experiences a rapid expansion when the negative-going driving pressure overcomes the static and the Laplace pressure caused by surface tension. The bubble radius increases until the growth is counteracted, typically by a return of the external pressure to positive values. After that the bubble implodes heavily. Such a process is called inertial cavitation, which is the most interesting cavitation regime from the application point of view. The critical value $P_{B}$ for entering the inertial cavitation regime is:

(13) $$P_{B}=p_{\mathrm{eq}}\left(1+{\left(\frac{4}{27}\frac{S^{3}}{1+S}\right)}^{1/2}\right),$$

where $p_{\mathrm{eq}}$ is the liquid pressure at the bubble wall if the bubble is at rest or, in other words, it is the ambient pressure. Quantity $S=2\sigma /p_{\mathrm{eq}}R_{\mathrm{eq}}$ is the dimensionless Laplace tension.

### 2.3 Wave propagation and attenuation

**Fig. 2:**
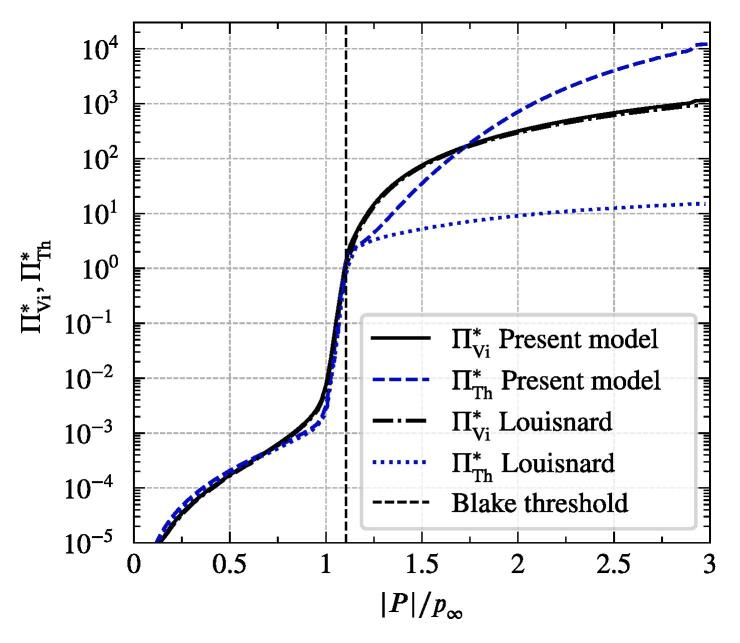
Dimensionless damping functions due to thermal and viscous dissipation for $R_{\mathrm{eq}}=3\;\mu m$ at 20 kHz from present model compared to Louisnard [28].

As mentioned above, for sake of efficiency of our macroscopic approach, we consider wave propagation in the frequency domain, which is governed by the Helmholtz equation:

(14) $$\nabla^{2}P+k^{2}P=0,$$

where *k* is the complex wave number, which will be discussed below. *P* is the complex pressure amplitude linked to the acoustic pressure in the time domain *p* by

(15) $$p=\frac{1}{2}\left(Pe^{i\omega t}+\overset{‾}{P}e^{-i\omega t}\right),$$

where $\overset{‾}{P}$ is the complex-conjugate of *P* and ω is the angular frequency of the acoustic source. The attenuation of the acoustic field due to cavitation bubbles is introduced by the wave number *k*. The most popular approach was proposed by Commander and Prosperetti [10], which assumes the bubble oscillations to be linear and, therefore, is called linear dispersion model. It makes use of the Caflisch equations [7], which describe wave propagation in a bubbly liquid in the time domain. The assumption of linearly oscillating bubbles is also the main drawback of this model, since most of the acoustic energy in cavitating liquids is dissipated by non-linearly (or better known as inertially) oscillating bubbles. This important aspect was addressed by Louisnard [28]. He provided terms describing the energy transferred to the surroundings by a single bubble over an acoustic cycle analyzing the solution of the ODE equations presented above in section 2.1:

(16) $$\Pi_{\mathrm{Vi}}=\frac{1}{T}\int_{0}^{T}16\pi \mu R{\overset{˙}{R}}^{2}dt,$$

(17) $$\Pi_{\mathrm{Th}}=\frac{1}{T}\int_{0}^{T}p_{g}\overset{˙}{V}dt,$$

where *T* is the time period of oscillations. Fig. 2 shows the two damping terms as functions of acoustic pressure amplitude, where we use the setup from Louisnard [28] and compare his results to the present model. The functions are nondimensionalized as follows:

(18) $$\Pi_{\mathrm{Vi},\mathrm{Th}}^{*}=\frac{\Pi_{\mathrm{Vi},\mathrm{Th}}}{p_{\infty }\frac{4}{3}\pi R_{\mathrm{eq},0}^{3}\omega },$$

with $R_{\mathrm{eq},0}$ being the equilibrium radius at rest (noted by 0). The comparison shows good agreement for the attenuation caused by viscous forces, whereas the thermal dissipation grows faster above the Blake threshold in the present model. Our investigations did not identify a specific reason for the deviation. We suspect that it is due to the differences in computation of material properties, where several molecular dynamics models may be used. Apparently a higher sensitivity of the thermal coefficient on the model details exists. However, since $\Pi_{\mathrm{Vi}}$ is still dominant for $|P|/p_{\infty }$ up to 1.5 for all considered $R_{\mathrm{eq}}$, we do not expect large deviations in the final results.

The dissipation terms are linked to the Helmholtz Eq. (14) through the real and imaginary parts of the squared wave number using the Caflisch et al. [7] model. We refer to the original paper [28] for the details of the derivation and provide only the resulting coupling:

(19) $$R\left(k_{\mathrm{cav}}^{2}\right)=\frac{\omega^{2}}{c^{2}}+\frac{3\beta \omega^{2}}{R_{\mathrm{eq},0}^{2}(\omega_{0}^{2}-\omega^{2})},$$

(20) $$I\left(k_{\mathrm{cav}}^{2}\right)=\frac{1}{-2\rho \omega V_{C}}\underset{i}{\sum }\frac{\Pi_{\mathrm{Vi},i}+\Pi_{\mathrm{Th},i}}{|P_{i}{|}^{2}},$$

with β being volume fraction of bubbles, also called void fraction. Since the present model considers discrete bubbles, the imaginary part of $k_{\mathrm{cav}}^{2}$ (20) is a sum of attenuation contributions from all of the bubbles in a control volume $V_{C}$. Quantity $P_{i}$ is the acoustic pressure amplitude at the bubbles’ position. Note that the real part equates to the one provided by the linear dispersion model [10]. According to Louisnard [28] the contribution of $R(k^{2})$ to $k^{2}$ is small and therefore the introduced error is negligible. The resulting damping described by $I(k^{2})$ is several orders of magnitude higher than in case of the linear dispersion model.

An additional condition is introduced to account for the fact, that cavitation inception takes place above the Blake threshold pressure. Note that the wave number in (19), (20) describes a case with the fully evolved cavitation. The link to $k^{2}$ from (14) is equivalent to the condition provided in [28] and reads

(21) $$k^{2}=\left\{\begin{array}{cc} R\left(k_{\mathrm{cav}}^{2}\right)+iI\left(k_{\mathrm{cav}}^{2}\right)\; & \mathrm{if}|P|>P_{B}\\ \frac{\omega^{2}}{c^{2}}\; & \mathrm{if}|P|⩽P_{B} \end{array}\right)$$

### 2.4 Bubble motion

We model cavitation bubbles as Lagrangian parcels which are accelerated corresponding to the following force balance:

(22) $$\langle m_{b}\rangle_{T}\frac{dU_{b}}{\mathrm{dt}}=F_{G}+F_{\mathrm{Am}}+F_{D}+F_{\mathrm{Bj}},$$

where $\langle \cdot \rangle_{T}$ denotes a quantity averaged over one acoustic time period *T* and $m_{b},U_{b}$ are bubble’s (gas) mass and velocity relative to the liquid velocity. The four forces on the right hand side are described in the following. The gravitational force is

(23) $$F_{G}=\left(1-\frac{\rho_{l}}{\rho_{b}}\right)\langle m_{b}\rangle_{T}g,$$

where g is the gravitational acceleration.

If a bubble accelerates or decelerates, it deflects some volume of the surrounding liquid. This volume motion is described by a force, which affects both, the dispersed and the liquid phase. The flow is assumed to be irrotational. According to Auton et al. [2], in the Lagrangian frame of reference and referred to a cavitation bubble, the force is described by

(24) $$F_{\mathrm{Am}}=C_{\mathrm{Am}}\rho \langle V_{b}\rangle_{T}\left(\frac{DU_{c}}{Dt}-\frac{dU_{b}}{dt}\right),$$

where $U_{c}$ is the carrier velocity, which might be slightly different from the liquid velocity depending on the models used (see (32)). Coefficient $C_{\mathrm{Am}}$ is computed by the potential theory and in the case of a sphere equals 0.5. The bubble velocity in the Lagrangian frame is only dependent on time. The corresponding derivative in (24) simplifies to the partial one and the final result is

(25) $$F_{\mathrm{Am}}=\frac{1}{2}\rho \langle V_{b}\rangle_{T}\left(\frac{\partial U_{c}}{\partial t}+U_{c}\cdot \nabla U_{c}\right)-\frac{1}{2}\rho \langle V_{b}\rangle_{T}\frac{dU_{b}}{dt},$$

where the last term includes the bubble’s acceleration, which is brought to the left hand side of (22). In this way, the force adds an additional (virtual) mass of $\frac{1}{2}\rho \langle V_{b}\rangle_{T}$ to the bubble. A certain error is introduced here by decoupling the average of the bubble volume from the bubble velocity (see Krefting et al. [21]), which is neglected in the following.

The primary Bjerknes force describes the influence of the acoustic waves on a cavitation bubble which is formulated in the general form:

(26) $$F_{\mathrm{Bj}}=\langle V_{b}\nabla p\rangle_{T}\text{.}$$

Since our approach considers *p* in the frequency domain via *P* and $V_{b}$ in the time domain, the computation of (26) is not obvious. Mettin [34] partially decoupled the two domains by dividing $F_{\mathrm{Bj}}$ into the contributions from standing and travelling waves. While the pressure amplitude gradient could be computed in the frequency domain, the phase of the pressure amplitude ϕ was directly linked to the bubble volume evolution. This issue was addressed by Louisnard [28]. The final formula is better readable if represented in index notation:

(27) $$F_{\mathrm{Bj},i}=\frac{\partial P}{\partial x_{i}}\left(I_{C}\cos\left(\phi -\psi_{i}\right)+I_{S}\sin\left(\phi -\psi_{i}\right)\right),$$

with $\psi_{i}$ being the phase of the pressure amplitude gradient and $I_{C},I_{S}$ are parameters, which are computed in a similar manner to the damping coefficients (16) and (17):

(28) $$I_{C}=\frac{1}{T}\int_{0}^{T}V_{b}\cos(\omega t)dt,$$

(29) $$I_{S}=\frac{1}{T}\int_{0}^{T}V_{b}\sin(\omega t)dt\text{.}$$

**Fig. 3:**
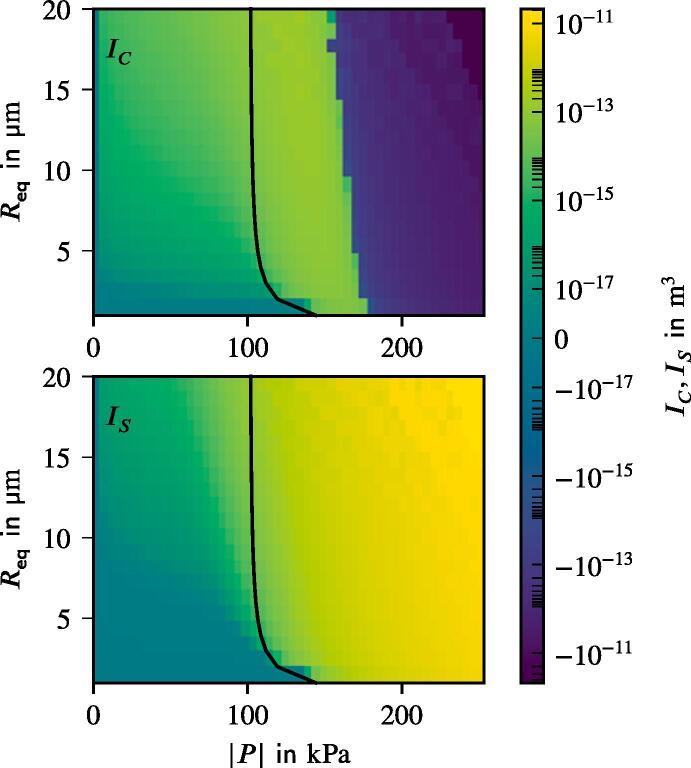
Contributions from the standing ($I_{C}$, top) and travelling ($I_{S}$, bottom) wave to the primary Bjerknes force. The black lines denote the Blake threshold. The legend is given in logarithmic scale apart of a linear region between −10^−17^ m^3^ and 10^−17^ m^3^.

These represent contributions from the standing ($I_{C}$) and travelling wave ($I_{S}$) to $F_{\mathrm{Bj}}$. The dependence on both $R_{\mathrm{eq}}$ and |P| is shown in Fig. 3. Note that $I_{S}$ is always positive in the given range. Both contributions exhibit a steep rise of several orders of magnitude at the Blake threshold. In fact, the bubbles experiencing a violent collapse during oscillations expose very different cross-sections to the wave in compression phase compared to the expansion phase, which is the actual cause of the primary Bjerknes force. The map for $I_{C}$ in Fig. 3 shows a force reversion at higher pressure amplitudes, which has been described in the literature previously by Akhatov et al. [1] and Matula et al. [31].

Magnaudet and Legendre [30] theoretically studied drag of a bubble with a time-varying radius. The authors concluded that it is twice as large as the Stokes’ one:

(30) $$F_{D}=12\pi \mu R_{T}\left(U_{c}-U_{b}\right)\text{.}$$

The formula applies for both low and high Re limits. A condition which has to be additionally satisfied concerns the Reynolds number defined as

(31) Reb=2R|R˙|ν

and restricts it to Reb≫1. The condition is satisfied when the bubble experiences the inertial cavitation regime for most of the time of an acoustic cycle.

We also account for the turbulent dispersion of the bubbles according to Gosman and Ioannides [17]. The model is a type of discontinuous random walk [6] or also known as eddy interaction model [18]. It interacts with the turbulence model from the Eulerian phase, estimates an additional fluctuating velocity U′ and alters the velocity seen by the bubble in the current cell to:

(32) $$U_{c}=U+U^{\prime },$$

where U is the velocity of the Eulerian phase. $U^{\prime }$ is a random value from the Gaussian probability distribution with a standard deviation being $\sqrt{2k/3}$, where *k* is the kinetic turbulent energy.

### 2.5 Fluid dynamics

The turbulent motion of the continuous (or Eulerian) phase is governed by the Unsteady Reynolds Averaged Navier Stokes (URANS) equations with an additional source term to account for the momentum transfer due to movement of the cavitation bubbles. We provide the relevant equations used in the model for the sake of completeness and also to show how the coupling with the Lagrangian phase is achieved: The continuity and the momentum equations for an incompressible Newtonian fluid are

(33) ∇·U=0,

(34) $$\frac{\partial U}{\partial t}+\left(U\cdot \nabla \right)U=-\frac{1}{\rho }\nabla p+\nu \nabla^{2}U+\frac{1}{\rho }S_{b}\text{.}$$

Here, U denotes the Reynolds averaged velocity, ρ the fluid density and *p* the pressure. The effective viscosity ν includes the molecular viscosity as well as the eddy viscosity due to turbulence. The last term is responsible for the coupling between the flow and the bubble motion. For a control volume $V_{C}$, in the finite volume method (FVM) being a single cell volume, the term reads [52], [43], [9]:

(35) $$S_{b,V_{C}}=-\frac{1}{V_{C}}\sum_{i}\langle m_{\mathrm{eff},i}\rangle_{T}\frac{\Delta U_{b,i}}{\Delta t},$$

where index *i* indicates a single bubble, $m_{\mathrm{eff}}=m_{b}+\frac{1}{2}\rho \langle V_{b}\rangle_{T}$ is the effective mass consisting of the interior gas and added masses. Quantity $\Delta U_{b,i}$ is the velocity difference of the bubble during the time interval Δt. The term accounts for the total change of the bubble’s momentum and transfers this change to the cell the bubble is in. We use a *k*-ω-SST model [33] to account for the turbulence.

### 2.6 Bubble population

The physics of the nucleation process is not yet fully clarified. This imposes an additional problem for modeling regarding the initial and boundary conditions of the bubble population. We carried out several calculations to test which assumptions are the most appropriate and concluded the following. Initial condition: the spatial bubble distribution is homogeneous in the whole computational domain with a specified void fraction $\beta_{0}$. The boundary conditions are:

• Walls and free surfaces: bubbles leave the domain undisturbed, which is equivalent to bubbles’ death and means that these bubbles are deleted from the calculation. At a rigid wall, if the size of a bubble is lower than the resonant size (which is true for all our setups), then it will jet towards the wall. At a free surface, a gas bubble outgases. The above condition satisfies both types of boundaries.
• Acoustic source (e.g. sonotrode): in the adjacent cells, the void fraction β is kept constant. This is done by introducing new bubbles if β falls below a specified threshold. The death condition is also present here for the case when a bubble touches the surface of the acoustic source.

**Fig. 4:**
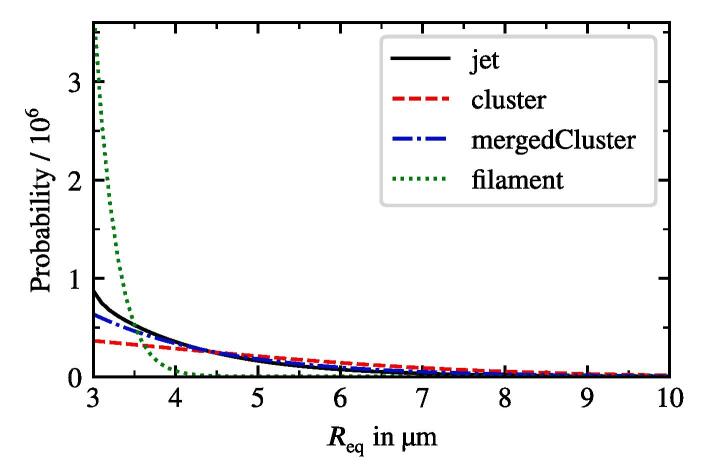
Bubble size distribution for different cavitation structures from [49].

Apart from the monodisperse populations, we incorporate bubble size distributions from experiments. Reuter et al. [49] studied a water jet entering a tank filled with water and sonicated at 27.5kHz. The authors evaluated bubble size distributions shown in Fig. 4 of the jet and other cavitation structures such as filament, cluster and merged cluster.

## 3 Implementation

The computation process consists of two parts. The radial bubble dynamics model presented in 2.1 is implemented in python [47] and is available online [25]. The system of equations consisting of (1), (2), (4) is non-dimensionalized by suitable quantities, e.g. initial equilibrium bubble Radius $R_{\mathrm{eq},0}$, initial gas temperature $T_{0}$ etc. The system is stiff in both dimensional and non-dimensional forms. Especially, in case of pressure amplitudes higher than the Blake threshold, the solvers of explicit Runge–Kutta class fail. The best performing solver is LSODA, which automatically switches between Adams and backward differentiation formula method [44].

On the other hand, the field equations including Helmholtz equation, fluid dynamics and bubble tracking are solved within the foam-extend software [53], which is based on the OpenFOAM technology utilizing the finite volume method. The implemented solver is available online [24]. Due to lack of complex numbers support in foam-extend, the Helmholtz equation is split into the real and the imaginary parts. Then, the equations are discretized and inserted in a block-coupled matrix solver [8], which enables implicit coupling between the equations. The approach is explained in detail in [26], where a linear damping model was used with a constant wave number *k*. In case of the non-linear damping presented in 2.3, the wave number depends on *P* and thus renders the Eq. (14) to be highly non-linear and requires an iterative approach. An additional numerical stiffness arises due to steep increase of $k^{2}$ around the Blake threshold as can be seen in Fig. 2. Relaxation or Picard iteration procedures fail to converge for most setups we tested. Therefore, we implemented a damped Newton–Raphson method with a polynomial line search following Dennis and Schnabel [14, pp. 116–119].

For further discussion, we introduce the Helmholtz Eq. (14) split into the real (subscript r) and imaginary (subscript i) parts. We substitute the squared wave number $k^{2}$ from (14) by *K* for simplicity and present the equation as a multi-dimensional function *F*:

(36) $$F(P_{r},P_{i})=\left[\begin{array}{cc} \nabla^{2}+K_{r} & -K_{i}\\ K_{i} & \nabla^{2}+K_{r} \end{array}\right]\left[\begin{array}{c} P_{r}\\ P_{i} \end{array}\right]=0\text{.}$$

The corresponding Jacobian is

(37) $$J=\left[\begin{array}{cc} \nabla^{2}+K_{r}+\frac{\partial K_{r}}{\partial P_{r}}P_{r}-\frac{\partial K_{i}}{\partial P_{r}}P_{i} & \frac{\partial K_{r}}{\partial P_{i}}P_{r}-K_{i}-\frac{\partial K_{i}}{\partial P_{i}}P_{i}\\ \frac{\partial K_{r}}{\partial P_{r}}P_{i}+K_{i}+\frac{\partial K_{i}}{\partial P_{r}}P_{r} & \nabla^{2}+K_{r}+\frac{\partial K_{r}}{\partial P_{i}}P_{i}+\frac{\partial K_{i}}{P_{i}}P_{r} \end{array}\right]$$

Then the Newton–Raphson method for an iteration *m* reads

(38) $$J\left(P_{r,m},P_{i,m}\right)\left[\begin{array}{c} \delta P_{r,m}\\ \delta P_{i,m} \end{array}\right]=-F\left(P_{r,m},P_{i,m}\right),$$

(39) $$\left[\begin{array}{c} P_{r,m+1}\\ P_{i,m+1} \end{array}\right]=\left[\begin{array}{c} P_{r,m}\\ P_{i,m} \end{array}\right]+\lambda \left[\begin{array}{c} \delta P_{r,m}\\ \delta P_{i,m} \end{array}\right],$$

where the last term prefixed by δ stands for the step in Newton direction and λ is a damping coefficient, which is discussed later. The solution of (39) requires the evaluation of the Jacobian *J* from (37) at iteration *m*. The four partial derivatives from this equation are unknown and analytical expressions cannot be provided due to the highly non-linear calculation procedure of $k^{2}$ (see (19), (20)). We use numerical differentiation, namely finite differences, to compute these terms. As an example, for the computation of

(40) $${\left(\frac{\partial K_{i}}{\partial P_{r}}\right|}_{m}=\frac{K_{i}(P_{r,m}+\Delta P_{r})-K_{i}(P_{r,m})}{\Delta P_{r}},$$

Eq. (20) and consequently (16) and (17) need to be evaluated, whereby input and initial values of the bubble radial dynamics ODE system are kept constant. Only $P_{r}$ is perturbed by a suitable $\Delta P_{r}$. The illustration makes clear that the evaluation of $K_{i}$ and $K_{r}$ is a computationally expensive task since it requires solution of the ODE system for at least one acoustic time period. Additionally, the quantities are computed for every individual bubble (in our case parcel of bubbles) since every bubble in the domain has a different equilibrium radius (in a polydisperse case) and is subject to different acoustic pressure amplitude *P*. In order to reduce the workload, we compute $\Pi_{\mathrm{Th}}$ and $\Pi_{\mathrm{Vi}}$ using the bubble radial dynamics code [25] for a range of *P* and $R_{\mathrm{eq},0}$ prior to the actual calculation and save the results as two dimensional interpolation tables. These tables are then used by the FVM code to obtain $K_{i}$ and $K_{r}$ by interpolation. This procedure increases the performance significantly.

The damped Newton–Raphson method introduces a damping coefficient λ, which equals 1 if the residual of the discretized Helmholtz equation system decreases and, thus, the full Newton–Raphson step is satisfactory. In the opposite case, i.e. if the residual increases, we use a backtracking algorithm (polynomial line search), which decreases λ successively until a residual reduction is achieved. Our experience showed that, eventually, λ may drop to very low values, e.g. on the order of $10^{-5}$, and the solver dwells at this damping level for many thousands of iterations. We address this issue by setting a lower bound for the damping coefficient $\lambda_{l}$. If the bound is reached, a recovery procedure is applied, during which we increase λ, apply a Newton–Raphson step while accepting residual growth. Then, the solver proceeds from the start point of the method, repeating recovery if necessary. This procedure keeps the computational effort at an acceptable level. All the calculations converged within 1000 iterations.

The cavitation bubbles are represented by the Lagrangian phase. In FVM, it is a common practice to track several bubbles with identical properties as a single instance, called parcel. We apply this approach in such a manner that the bubble size distribution and the spatial coverage of bubbles remain comparable to a setup, where a single bubble per parcel is used. Additional quantities, which depend on $R_{\mathrm{eq},0}$ and *P* are required for the force balance (22), namely $\langle R\rangle_{T},\langle m_{b}\rangle_{T},I_{C},I_{S}$, and thus are introduced as 2D interpolation tables in a similar way as $\Pi_{\mathrm{Th}}$ and $\Pi_{\mathrm{Vi}}$. The fields ϕ and $\psi_{i}$ from (27) are computed in the Eulerian phase. These and all other quantities required in the Lagrangian phase at the parcel’s position are spatially interpolated from the Eulerian one.

## 4 Setup with a 12 cm sonotrode

### 4.1 Verification

**Fig. 5:**
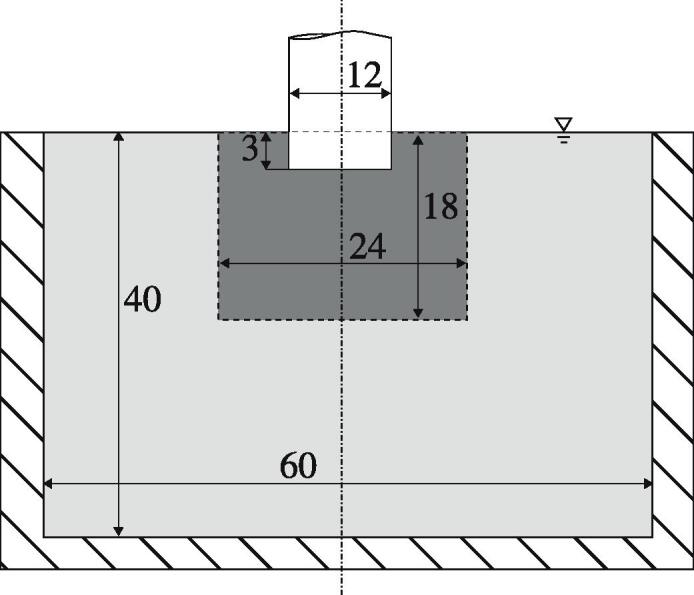
A geometry for the model verification (light gray area) and a geometry for the investigation of the cavitation flow denoted by the dashed line (dark gray area). The dimensions are given in cm.

**Fig. 6:**
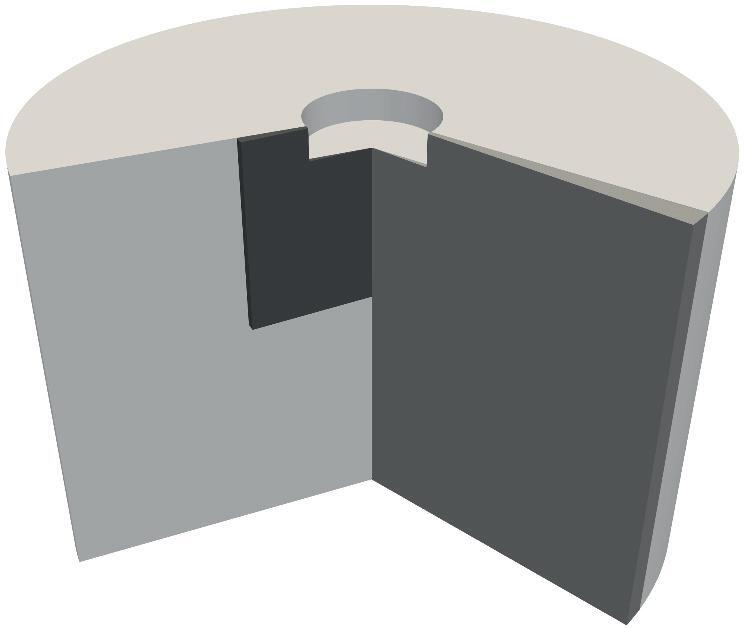
Illustration of the axisymmetric and the corresponding wedge geometries for the model verification (gray) and for the Euler–Lagrange calculations (dark gray).

In order to verify our implementation of the non-linear damping model, we use the same geometry as Louisnard [29], who borrowed it from Moussatov et al. [38], whereby modifying it from rectangular to cylindrical (axisymmetric) form. Fig. 5 shows a sketch of the cylindrical tank with 40 cm depth and 60 cm diameter filled with water. The sonotrode of 12 cm diameter is immersed down to 3 cm below the free surface. We decrease the computational effort by modelling a wedge of 5° instead of the full 3D geometry as depicted in Fig. 6. Louisnard [29] used a monodisperse bubble distribution that was also spatially homogeneous without accounting for the inception condition (21), which renders it inhomogeneous. We reproduce these constraints by introducing a monodisperse bubble population spread evenly in space with $R_{\mathrm{eq}}=2\;\mu m$ and by running the computation for a single time step without considering the primary Bjerknes force. In this way, the computation is stationary. The liquid properties are: ρ = 1000 kg/m^3^, c = 1500 m/s, μ = 10^−3^ Pa s, σ = 0.0725 N/m and $p_{\infty }$ = 101.3 kPa. The acoustic frequency of the sonotrode is 20kHz and the void fraction is set to $\beta =1.206\cdot 10^{-5}$. The boundary conditions for both, acoustics and fluid dynamics are summarized in Table 1, where n represents a unit normal vector of the corresponding boundary. The pressure gradient at the sonotrode-liquid boundary is computed in the solid with the finite element program COMSOL Multiphysics [11], where a part of the sonotrode is modeled and a solid mechanics solution in frequency domain is obtained. The vertical displacement amplitude of the sonotrode is set to $d_{0}=1.4\;\mu m$. Note that the sonotrode bottom and lateral wall vibrate in antiphase, which is reflected by the signs of the corresponding pressure gradients in Table 1. In our experience, this is crucial for the structure of the acoustic pressure field in the tank.

**Table 1:** Boundary conditions.

**Domain**	**Boundary**	**Condition**
Acoustics	Walls	Sound-hard: ∇P·n=0
	Free surface	Sound-soft: P=0
	Sonotrode bottom	∇P·n=32 MPa/m
	Sonotrode side	∇P·n=-9.5 MPa/m

Flow	Walls, sonotrode	∇p·n=0,U=0
	Free surface	Slip

Turbulence	Walls, sonotrode	Wall functions
	Free surface	Slip

We carried out a mesh convergence study. The area around the sonotrode was refined in order to resolve large gradients of acoustic pressure amplitude. The mesh chosen for calculations, which delivered results accurate enough and showed best performance, consists of 55,000 orthogonal hexahedra.

**Fig. 7:**
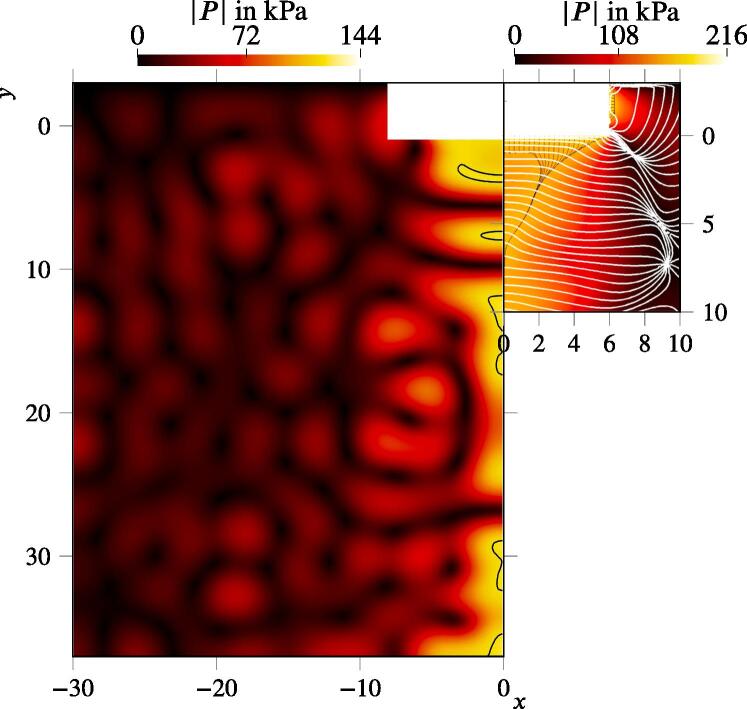
Field of acoustic pressure amplitude of the wedge test case from our calculation on the left and from Louisnard [29] on the right (reprinted with the permission from Elsevier). Black solid line represents Blake threshold. The dimensions ar.e given in cm.

Fig. 7 displays the pressure field. The areas where the Blake pressure threshold is exceeded are depicted with the black solid lines, which are seen as cavitation inception domains. The areas are located at and below the horn and are also present at the bottom of the tank. The field near the sonotrode looks similar to the one from [29] (Fig. 12 in the reference). The cone structure directly under the sonotrode is recognizable though it has a slightly different shape: There is a nodal zone at approximately. 5 cm beneath the horn and the high pressure zones are stretched in the radial direction. Louisnard [29] defined the bottom to be anechoic, which is sound-hard in our calculations. This may explain the small differences in the resulting acoustic pressure distribution, in particular, the standing wave features (nodal zones).

**Fig. 8:**
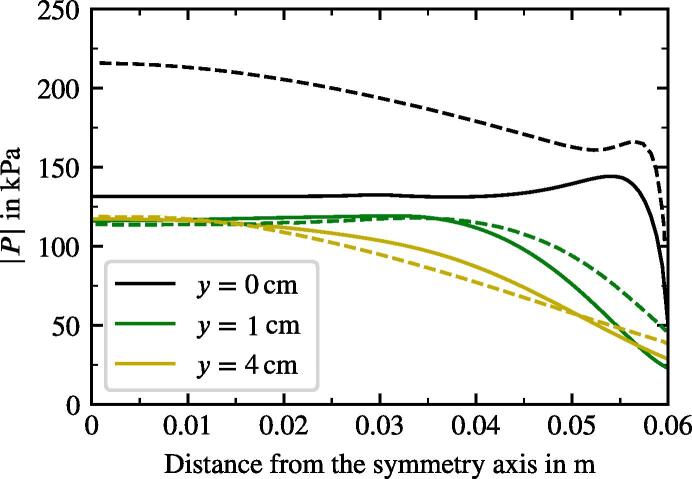
Radial distribution of acoustic pressure amplitude from this model (solid lines) and from Louisnard [29] (dashed lines) at distance *y* from the sonotrode surface.

We also compared the radial distribution of |P| at three vertical distances from the sonotrode bottom. The comparison presented in Fig. 8 demonstrates good agreement for 1 cm and 4cm distance with the values given in Fig. 10 of Ref. [29]. The values of |P| deviate in the outer region by several kPa only. The pressure amplitude directly at the sonotrode differs by up to 100 kPa. Several issues can explain the deviations. First, we impose a pressure gradient at the sonotrode whereas Louisnard [29] carries out a simulation directly coupled with solid mechanics. Second, the non-linear damping of the present model is higher due to our computations (see Fig. 2). Ultimately, the agreement in the regions of interest (around the sonotrode) is reasonable.

### 4.2 General overview of the flow

For transient computations, we shrink the geometry to the region depicted by the dashed line in Fig. 5, which means that the new geometry is 18 cm tall with a 24 cm diameter. This is done for the sake of computational efficiency, whereby the characteristic features of the pressure field are preserved as discussed below. The boundary conditions correspond to those listed in Table 1, whereby the outer lateral side and the bottom of the new setup are walls. Initially, we define a case, which serves as a reference for the studies presented in the following sections. The initial and wall threshold void fraction (see Section 2.6) of the reference case is the same as in the verification test case: $\beta_{0}=1.206\cdot 10^{-5}$. The bubble population is prescribed according to the size distribution of a jet structure presented in Fig. 4. The bubble and flow velocities are initialized to zero. First, we analyze the acoustic pressure field shown in Fig. 10a. The conical structure under the sonotrode similar to the one from the larger geometry depicted in Fig. 7 is preserved. The two first antinodes from the latter seem to be merged into a single structure in Fig. 10a. The pressure distribution further in radial direction has also smaller amplitude and similar shape compared to the larger geometry. We point out that the maximum |P| is 160 kPa, but this value is reached only at the sonotrode bottom. The rest of the field exhibits lower values than the larger geometry. The reasons for this are discussed in the next section. From the analysis of the temporal development, we state that the acoustic pressure field stays quasi-stationary showing fluctuations only up to 10 kPa.

**Fig. 9:**
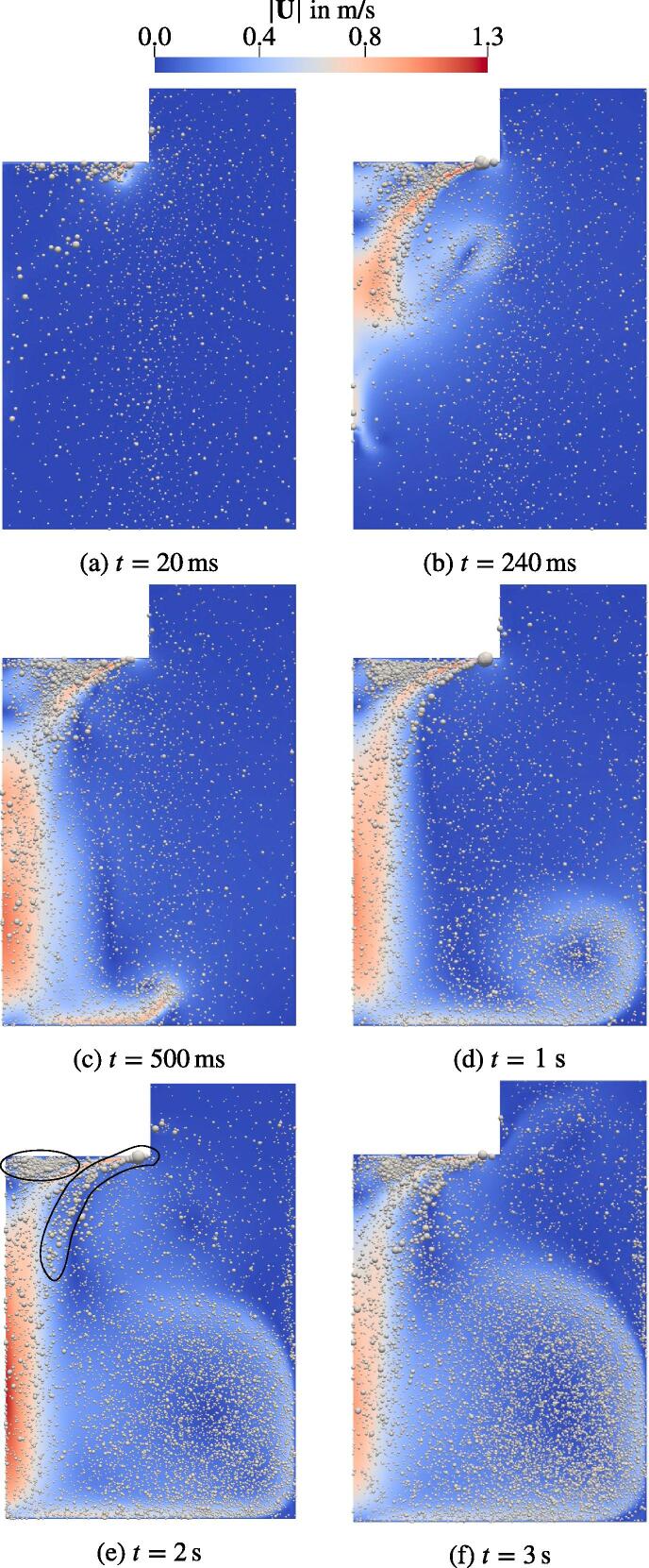
Time series of the flow velocity with bubbles with the start at t=0s. Every 100th bubble is presented. Bubble size is proportional to $\langle R\rangle_{T}$ and enlarged by factor 100.

**Fig. 10:**
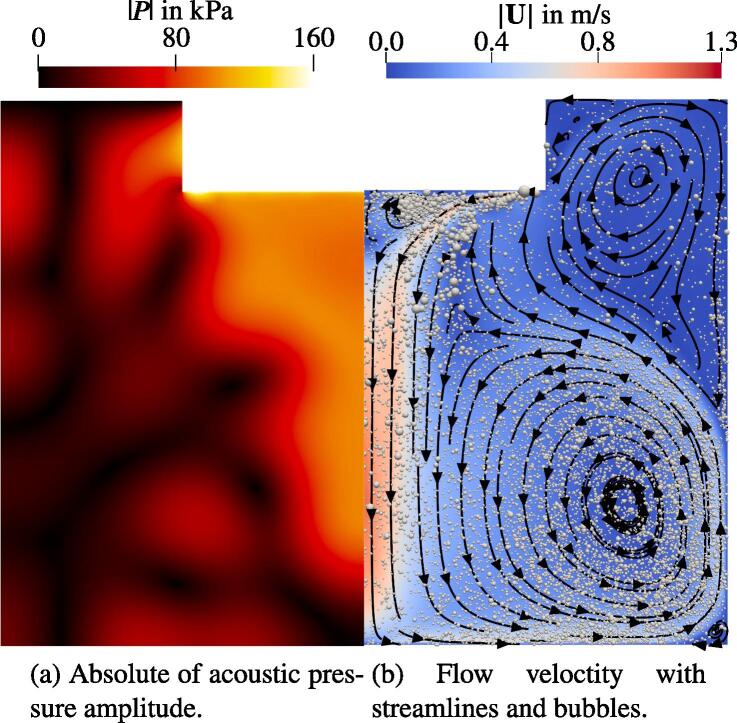
Acoustic pressure and streamlines for the reference case at t=3s.

Fig. 9 shows the velocity field with bubbles of the first 3 s. At 20 ms, a jet forms at the outer part of the sonotrode bottom. Then, the jet evolves forming a vortex at 240 ms and hitting the container bottom at around 500 ms. Three seconds after the start a quasi-stationary flow is obtained that persists until the end of the calculation at t=10s (not shown here). Two large vortices form, which are visible in Fig. 10b: a large one in the lower corner of the container and a smaller one in the upper part next to the sonotrode. A large part of the bubbles moves with the flow and dwells in the lower part of the tank. Further, there are two highly populated zones next to the sonotrode annotated in Fig. 9e. The first one is situated directly under the sonotrode and is clearly distinguishable after t=1s. A comparison to Fig. 10b reveals that this is due to a vortex situated directly under the sonotrode. The second one with a curved shape emerges at the outer boundary of the jet. From Fig. 10b we conclude that this is the area of shear flow appearing between the jet and the two larger vortices.

**Fig. 11:**
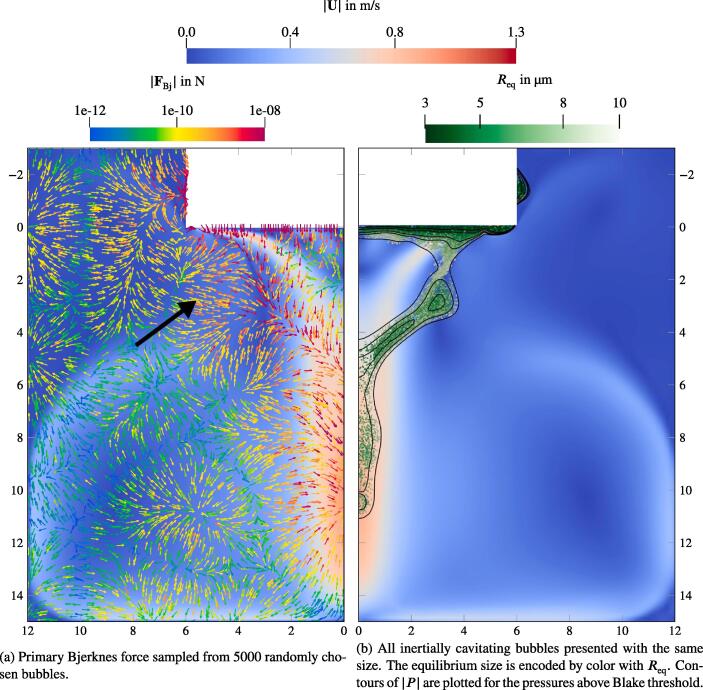
Velocity field with overlays for a fully developed flow. The dimensions are given in cm.

Note that we show every 100th bubble in Fig. 9. Each of these is scaled with the radius averaged over a single acoustic cycle $\langle R\rangle_{T}$. Thus, bubbles which cavitate in both regimes, stable (under the Blake threshold) and inertial (above the Blake threshold) are presented. This issue is addressed in Fig. 11b, which shows only inertially cavitating bubbles. The black contours correspond to isosurfaces of the Blake threshold pressures, beginning with $P_{B}=103.3\;\mathrm{kPa}$ for $R_{\mathrm{eq}}=10\;\mu m$ bubbles and ending with $P_{B}=111.9\;\mathrm{kPa}$ for the smallest bubbles of $R_{\mathrm{eq}}=3\;\mu m$. The difference between the thresholds for different bubble sizes is confirmed in Fig. 11b, where the bubbles with smaller $R_{\mathrm{eq}}$ are only present directly at the sonotrode (high |P|) and only larger bubbles cavitate inertially further downstream (lower |P|). In the same figure, we also observe that the conical part of the jet is shifted towards the symmetry axis compared to the region of the inertial cavitation. This deviation is also present at the early stage of the jet development, e.g. t=500ms. Thus, the shift is not caused by the vortex structure. During further investigation we examine the primary Bjerknes force shown in Fig. 11a. The bubbles from the region pointed out by the black arrow experience large Bjerknes forces around 10^−9^ N directed towards the central axis, whereas on the inner side of the cone structure at the same height the driving from the acoustic field is an order of magnitude lower and mostly directed downwards. In conclusion, the bubbles provide more momentum to the liquid towards the symmetry axis than into the opposite direction, which causes the shift of the jet structure.

### 4.3 Influence of population type

**Fig. 12:**
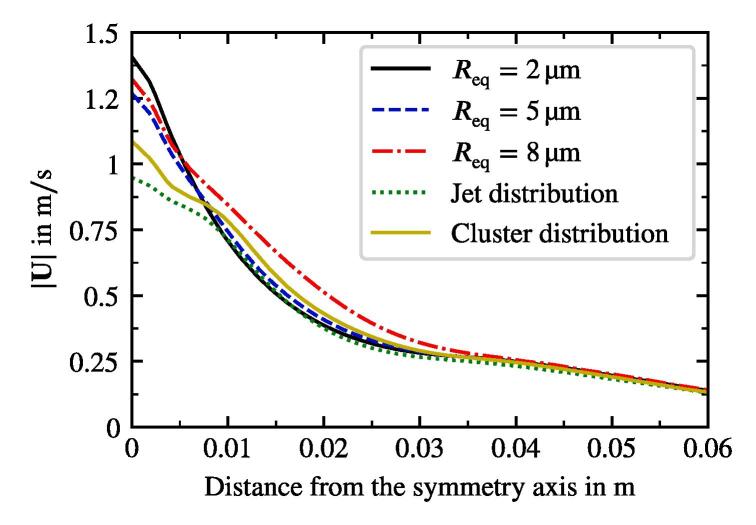
Radial velocity profiles of the jet at 9 cm under the sonotrode for three monodisperse cases and two distributions from Fig. 4. Velocity is averaged for several times around t=3s.

**Fig. 13:**
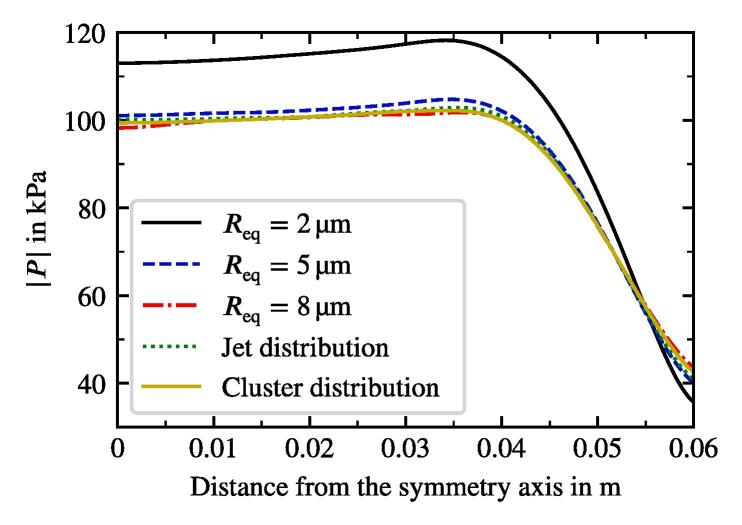
Radial profiles of the acoustic pressure amplitude at 1 cm under the sonotrode for three monodisperse cases and two distributions from Fig. 4. |P| is averaged for several times around t=3s.

**Fig. 14:**
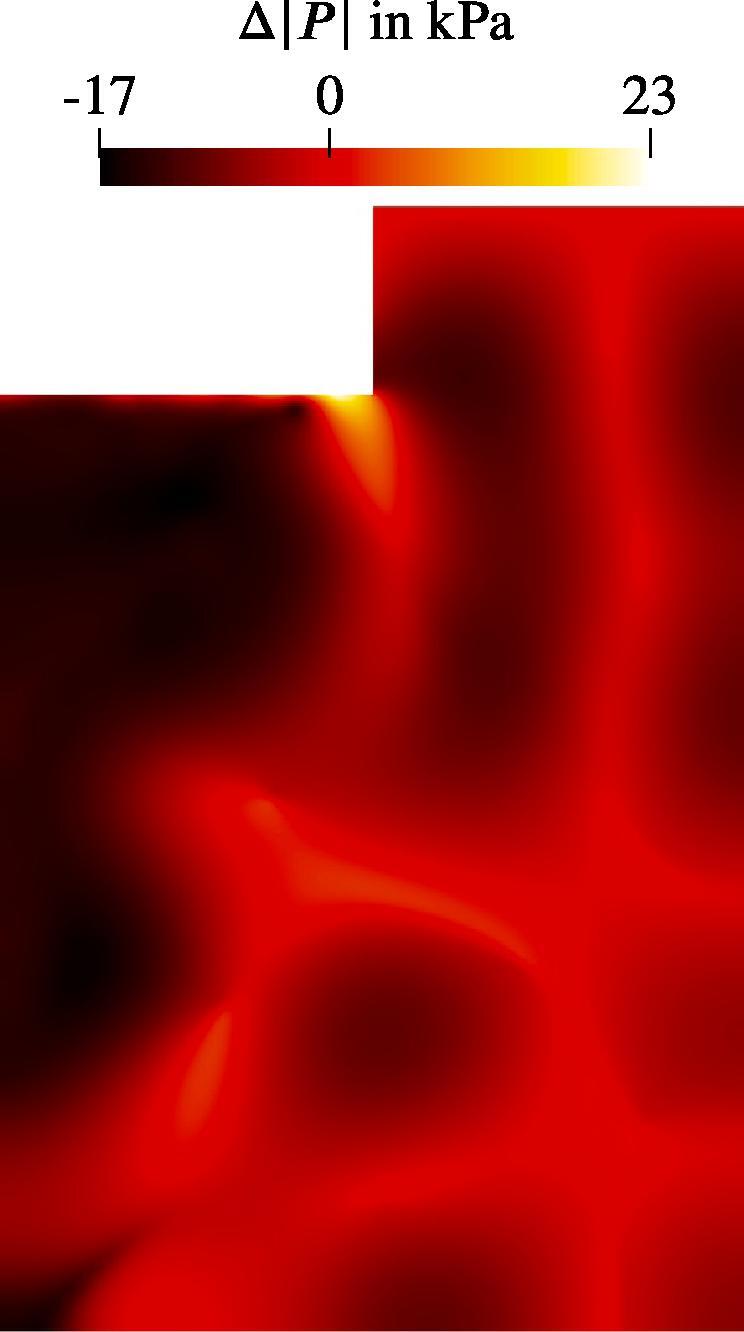
Difference in |P| between the reference case and the monodisperse case with $R_{\mathrm{eq}}=2\;\mu m$.

We study the influence of the bubble size distribution on the acoustic pressure and the flow velocity. The setup corresponds to that from the reference case defined previously. The only change affects the bubble size distribution. It is obviously uniform for the monodisperse cases. We also consider an additional distribution from Reuter et al. [49], which describes the population in a bubble cluster away from the jet (see Fig. 4). The results show, that the conical structure for both the flow and the acoustic pressure is preserved across all setups. Fig. 12 provides a comparison between the radial velocity profiles for the five cases in the region, where the flows exhibit the largest velocities. The greatest deviation takes place in the jet’s core region. The highest velocities are reached for the monodisperse cases, whereby the smallest $R_{\mathrm{eq}}$ causes the highest velocities. Reviewing the dependency of the primary Bjerknes force on the equilibrium bubble radius from Fig. 3, we deduce that for the same |P| smaller bubbles experience smaller $F_{\mathrm{Bj}}$. At first glance, this finding contradicts the observation from Fig. 12, but there are two major aspects to consider. First, the void fraction at the initialization and at the wall boundaries is the same for all cases. Therefore, there are more bubbles in the domain for a case with smaller equilibrium radius, which may cause a larger momentum contribution. Second, the |P| field differs between the cases as may be seen in Fig. 13. The highest values correspond to the Blake threshold pressure $P_{B}$, which is larger for smaller $R_{\mathrm{eq}}$ (see black line in Fig. 3). We confirm that the highest acoustic pressure amplitudes in the rest of the domain also match the Blake threshold. This is also the reason for qualitatively similar radial profiles of |P|: acoustic energy is dissipated at the horn up to the levels where the attenuation is several orders of magnitude lower and Fig. 2 suggests that these levels lie around $P_{B}$. To illustrate this point, we provide a difference pressure field in Fig. 14, where |P| of the 2 μm-monodisperse case is subtracted from that of the reference case. Almost all areas with the local maxima of |P| exhibit higher values for the 2 μm monodisperse case.

Interestingly, the polydisperse cases (jet und cluster distributions) in Fig. 13 exhibit maximum |P| values around the Blake threshold $P_{B}$ of the largest bubbles from the population, which is $R_{\mathrm{eq}}=10\;\mu m$. From this, we conclude that the size of the largest bubbles determines which maximum acoustic pressure may be reached. This observation also explains lower velocities of the polydisperse distributions at the center of the jet in Fig. 12. Primary Bjerknes force decreases severely, when the pressure drops below $P_{B}$ (see Fig. 3). The affected part of the population consists of smaller bubbles, because their Blake threshold is higher. As a result, they do not provide as much momentum as in the monodisperse cases and the flow velocity is lower.

### 4.4 Influence of void fraction

**Fig. 15:**
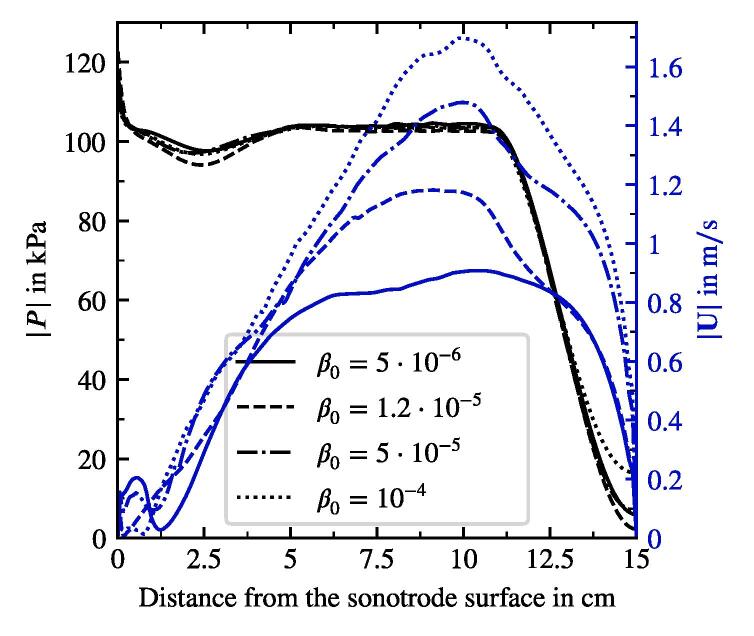
Acoustic pressure amplitude and velocity magnitude along the symmetry axis for the cases with different initial and boundary threshold void fraction $\beta_{0}$.

An important parameter which is rather hard to determine experimentally is the void fraction. We study its influence on the cavitation flow in the considered geometry. Four cases are setup with void fractions $\beta_{0}$ being 5 · 10^−6^, 1.2 · 10^−5^ (reference case), 5 · 10^−5^ and 1 ·10^−4^. Other parameters and boundary conditions correspond to the reference case. Mainly, the acoustic pressure and the velocity fields are examined. The shape of the former remains unchanged for all the cases. Only small deviations of |P| between the cases are present. Fig. 15 shows the profiles of |P| along the symmetry axis as black lines. In the region from 5cm and 11cm, where the tip of the conical structure is formed, we observe an acoustic pressure plateau with very small fluctuations along the axis. |P| fluctuates around the Blake threshold pressure for bubbles of $R_{\mathrm{eq}}$ between 8 and 10 μm. The behavior is similar to the situation described above during the study of the population type influence: The acoustic attenuation is the greatest near the sonotrode, which is confirmed by the steep acoustic pressure gradient at the figure’s origin. In this area the whole population contributes to the damping, whereas further downstream the attenuation from the largest bubbles is enough to keep |P| at the level around $P_{B}$. As a result, if a certain level of attenuation is achieved, which is the case for the lowest $\beta_{0}$, further increase in void fraction does not lead to more damping of acoustic pressure.

The blue lines in Fig. 15 describe the velocity magnitude along the symmetry axis. The peak values lie at a distance of around 10 cm from the sonotrode bottom. Velocity magnitudes exhibit large deviations up to 1 m/s difference. The trend is also clear: cases with higher void fraction show higher velocity. From the discussion above, we infer that this is not caused by differences of the acoustic pressure field between the cases. The cause is rather the void fraction itself, more precisely the bubble number. The cases with higher void fractions are populated with larger number of bubbles. We remind that in the present model the only source of the momentum in the liquid is bubble motion driven by acoustics. Since the |P| field is for all void fractions alike and thus the driving of bubbles is similar, just the higher bubble number leads to a greater momentum transfer to the liquid.

## 5 Setup with a 1 cm sonotrode

### 5.1 Calculations

#### 5.1.1 Setup

**Fig. 16:**
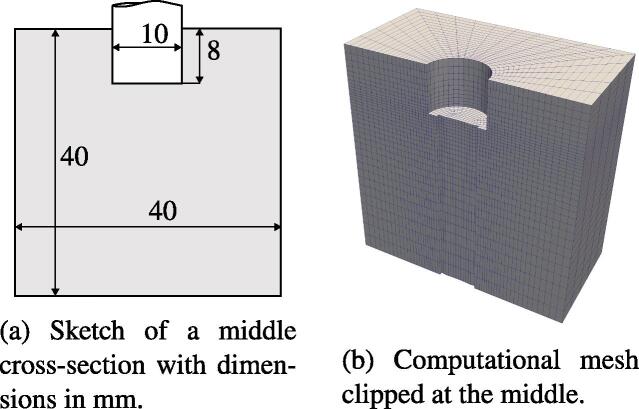
Setup of the cuvette with a 1 cm horn for numerical experiments.

The investigations above verify our model and quantify the influence of the parameters, which are rarely measured in experiments. In the following, we consider a third geometry in order to validate whether the bubble behavior seen in the experiments is predicted by the present model. The geometry stems originally from Nowak [39] who carried out experiments in a cubic cuvette. The main difference to the setup from Section 4 is the considerably smaller diameter of the horn being 1 cm in the present case. The experimental setup is transferred to a 3D computational geometry shown in Fig. 16a whereby assuming all solid parts to be boundaries. The cuvette does not exhibit a free surface. The lid, the bottom and one lateral wall are made of metal. The other walls are made of acrylic glass, which is considered to have the same impedance as water and therefore acoustic waves propagate unhindered from the interior of the cuvette to the glass-air boundary. Thus, it is assumed that these walls represent a sound soft boundary. The metallic walls are modeled as sound hard boundaries.

The acoustic behavior of the horn tip during excitations was investigated with a solid mechanics solver.^1^ An input displacement was introduced at the top of the horn tip and the response at its lateral wall and bottom were analyzed. The results show that the displacement amplitude of the sonotrode lateral wall is several orders of magnitude smaller than the one of the sonotrode bottom. This is confirmed for a range of input displacements. Therefore, no solid mechanics co-simulation for horn modelling is carried out during this study. The displacement amplitude is translated to the gradient of acoustic pressure and imposed directly at the sonotrode bottom, whereas the sonotrode lateral wall is assumed to be sound hard. Other boundary conditions correspond to those used in the 12 cm sonotrode case (see Table 1) including the water properties (Section 4). The acoustic frequency is set to 17.3 kHz in accordance with Nowak [39] and the initial bubble fraction is $\beta =1.10^{-5}$.

#### 5.1.2 Results

**Fig. 17:**
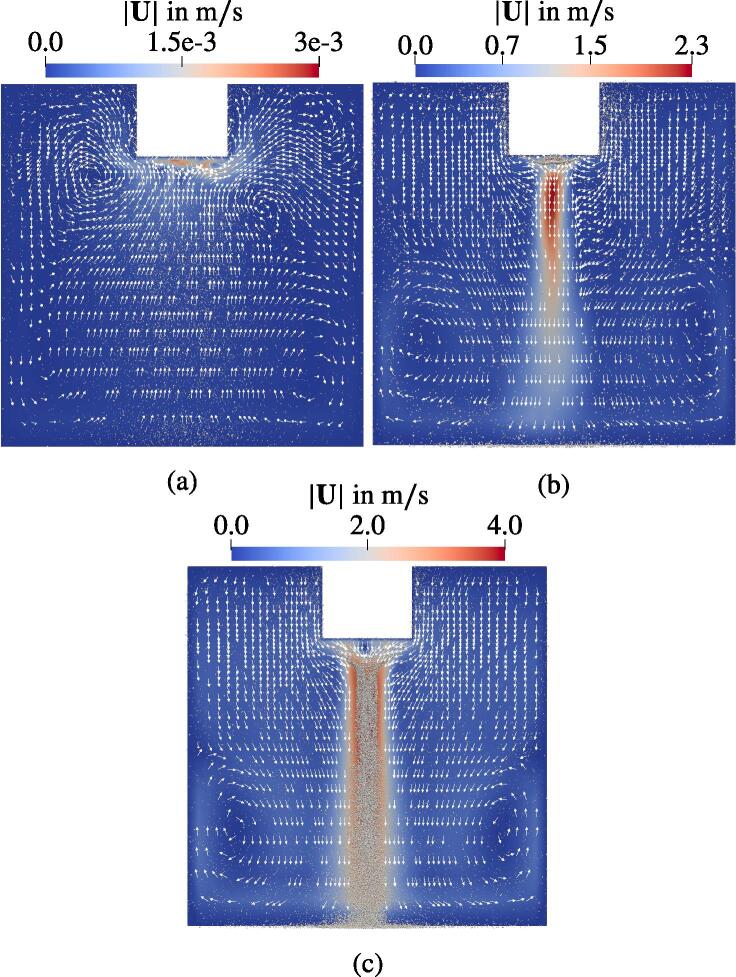
Flow fields for horn displacement amplitudes $d_{0}$ of 2μm (a), 5μm (b) and 10μm (c) with bubbles.

Fig. 17 gives an overview of the fluid velocity depending on the displacement amplitude of the sonotrode bottom. The white rectangular at the top of the images is the lower part of the sonotrode. Three displacement amplitudes of 2 μm, 5 μm and 10 μm are considered. Note that the legends are scaled differently, since the highest and the lowest velocities vary by several orders of magnitude. Further, the flows underneath the sonotrode in Fig. 17a and c point to different directions. The flow for $d_{0}=10\;\mu m$ is directed towards the bottom of the cuvette, whereas in the case of $d_{0}=2\;\mu m$, the liquid moves towards the sonotrode with low velocity. These two cases are considered in the following and the variant with $d_{0}=5\;\mu m$ will be discussed later.

**Fig. 18:**
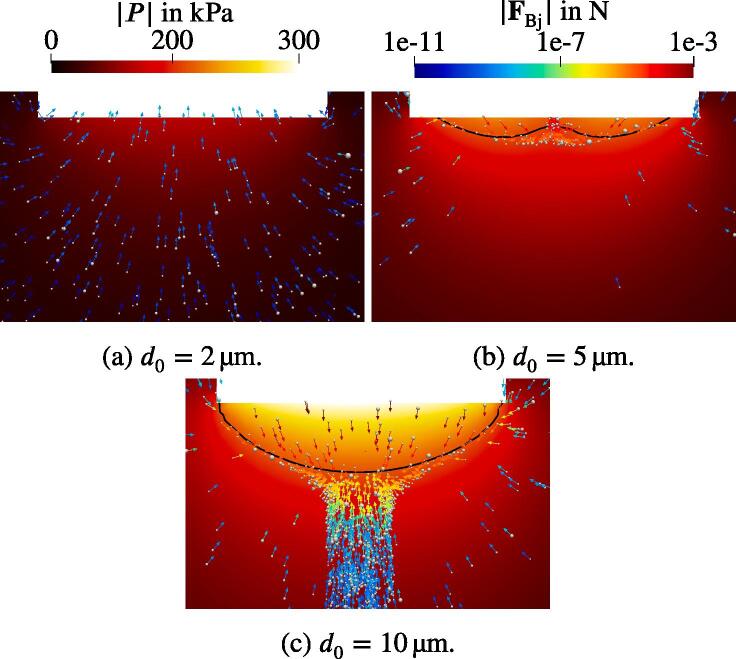
Comparison of the acoustic pressure field for different horn displacement amplitudes. Every tenth bubble with the corresponding primary Bjerknes force is presented. Black lines denote 160 kPa threshold.

These findings can be explained by studying the corresponding acoustic pressure fields in Fig. 18. A distinctive feature of the acoustic pressure field for $d_{0}=10\;\mu m$ is the fact that its amplitude exceeds 160 kPa. The latter is the approximate force reversion threshold of the standing wave contribution to the primary Bjerknes force $I_{C}$, which was presented in Fig. 3. It is denoted as Bjerknes threshold $P_{\mathrm{Bj}}$. According to the analysis by Akhatov et al. [1], the pressure antinodes attract and the pressure nodes repulse the bubbles if the pressure amplitude is below the threshold. For |P| higher than the threshold, they exchange the roles: The nodes exhibit attraction and the antinodes repulsion on the bubbles. For the studied bubble population, the lowest $P_{\mathrm{Bj}}\approx 160\;\mathrm{kPa}$ corresponds to the largest $R_{\mathrm{eq}}=10\;\mu m$. The bubble velocities confirm the theory of Akhatov et al. [1]: The horn bottom acts as an antinode and is, therefore, repulsive for the nearest bubbles, because the pressure amplitude reaches up to 270 kPa. Further away from the horn, where |P| drops below $P_{\mathrm{Bj}}$, the bubbles are attracted by the sonotrode. This is depicted by the arrows, which represent the primary Bjerknes force. Nonetheless, the momentum intake during the repulsion (in the area above the black line) is so high that the deceleration provided by the reversed Bjerknes force after the threshold (below the black line) does not reverse the bubbles but only reduces their velocities. Therefore, a bubble jet forms. In the case of $d_{0}=2\;\mu m$ the threshold $P_{\mathrm{Bj}}$ is not reached at any location and thus the sonotrode attracts the bubbles from the whole domain.

**Fig. 19:**
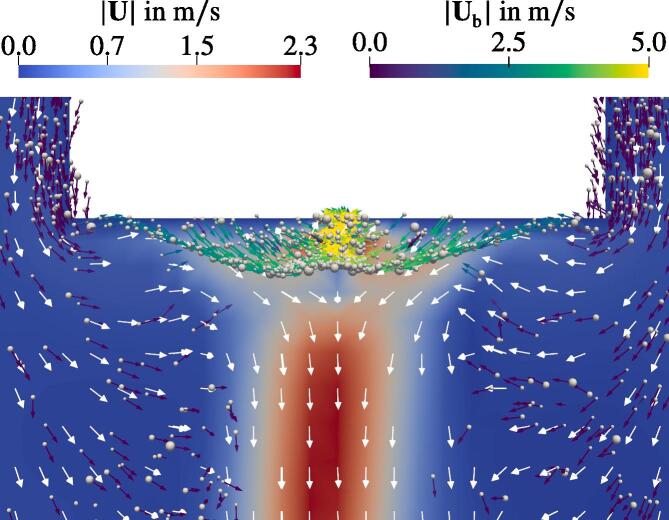
Flow field with the normalized velocity vectors (white arrows). The bubbles scaled with $10R_{\mathrm{eq}}$ and shown with velocity vectors $U_{b}$.

The Bjerknes threshold is exceeded for the intermediate displacement amplitude of $d_{0}=5\;\mu m$. It is located closer to the sonotrode than in the 10μm case as can be seen in Fig. 18. Comparing the two in Fig. 17 reveals that, first, the jet velocities differ approximately by factor two and the jet itself is populated with bubbles in case of the larger amplitude but it is free of bubbles for $d_{0}=5\;\mu m$. At a first glance, this is in contrast to the previous findings, where the jet is formed due to the motion of the bubbles. The bubble behavior is addressed in Fig. 19, where the region below the horn is magnified. Most of the bubbles emerging from the sonotrode surface travel several mm downwards and towards the center axis of the sonotrode, which is due to the shape of the Bjerknes threshold. At this location, they turn upwards and proceed to the horn surface, where they escape the domain according to the boundary condition explained in Section 2.6. The momentum is transferred from the bubbles to the liquid during this time. According to Eq. (35), the momentum transfer takes place when the bubbles accelerate. The highest acceleration occurs directly after the bubble injection at the horn surface. Eventually, a downward liquid jet forms pushing the bubbles, which were located further away from the horn at the initialization. Since the bubbles, which emerge at the horn, do not join the jet, it stays free of bubbles.

### 5.2 Experiments

#### 5.2.1 Setup

An in–house made exponential aluminium horn with a 10 mm diameter tip was submerged 1 cm deep vertically into de-ionized (DI) water in a cubic PMMA cell (6 cm edge length, 5 cm filling height). The driving frequency was 21 kHz. Observation from the side via a high-speed camera (Photron APX-RS 250K), coupled to a long-distance microscope (K2, Infinity), allowed to observe the cavitation bubbles and to measure the peak-to-peak displacement of the horn tip. The experiments were carried out at the normal surrounding temperature and pressure (NTP).

#### 5.2.2 Results

**Fig. 20:**
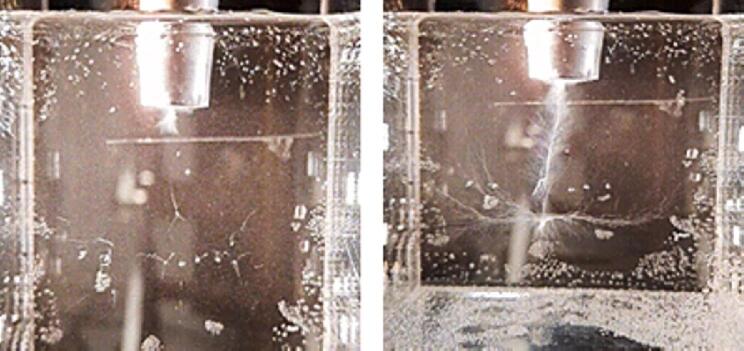
Typical bubble structures in DI water sonicated at 21 kHz with the sonotrode of 1 cm diameter (image width 5 cm). Left: low sonotrode displacement amplitude (2–5 μm); right: high sonotrode displacement amplitude (10–15 μm).

Here we show data for “low” (2–5 μm) and “high” (10–15 μm) sonotrode displacement amplitudes. Typical long-term exposures of the bubble patterns for both cases are presented in Fig. 20.

In each of the cases, a vertical “bubble jet” appears which touches the sonotrode tip. For lower amplitude, the jet is short, while it extends downwards for the higher amplitude. Apart from the jet, horizontal streamers of bubbles can be identified below. They gather around a standing wave pressure antinode, which is forming near the middle of the cuvette as a result of reflections from the walls.

**Fig. 21:**
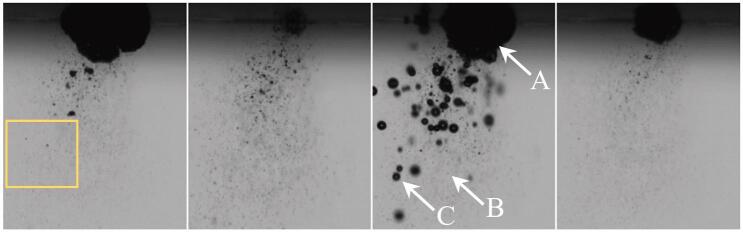
Short term exposures (1 μs) of the bubble jet at low driving amplitude. Interframe time is 1.4 T = 66.6 μs, image width is 2.48 mm. Large (A), small (B) and medium (C) bubbles are indicated by the arrows in the third frame.

**Fig. 22:**
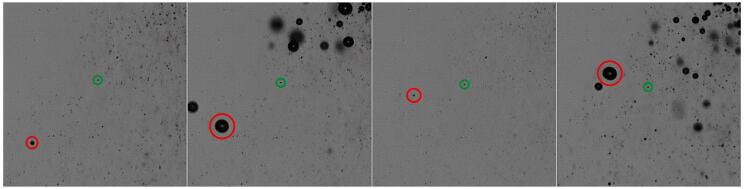
Tracking of individual bubbles under the sonotrode tip for small amplitude in the region of the yellow rectangle in Fig. 21 (width 1.14 mm). Red circles: medium sized bubble moving fast and upwards at 2.3 m/s; green circles: small bubble moving slowly downwards at 0.35 m/s. Interframe time 66.6 μs, exposure time. 1 μs.

Fig. 21 shows frames from a high-speed movie of the bubble jet at low driving amplitude. The representative short-term exposures exhibit individual bubbles and their varying expansion at different driving phases. Thus the volume oscillations of the bubbles can be perceived. Closer inspection of the high-speed recordings reveal mainly three types of bubbles with different sizes and behaviors: (A) A large bubble that attaches to the tip, oscillating with the maximum radius in the range of 50–300 μm. It undergoes surface distortions and instabilities, splitting off many small bubbles during its collapse. (B) These split-off bubbles define the second population. They are quite small with radius from a few micrometers to sub-micrometers (bubbly “mist”), and their volume oscillations are weak. They move rather slowly several millimeters downward with an average speed in the range of 0.3–0.5 m/s, away from the sonotrode tip. Some of these small bubbles grow larger on their way due to collisions and/or rectified diffusion. By increasing their size, they feed the third category of bubbles, (C). These medium-sized bubbles show large volume oscillations with a maximum radius variation in the range of 10–50 μm. They move again upwards, towards the large bubble and the sonotrode tip, with speeds in the range of 2–4 m/s. Thus they run much faster than the small bubbles and in opposite direction. These medium-sized bubbles can jump several 100 micrometers in one acoustic cycle and are often observed to undergo jetting in their collapse phase. The upward motion of such a bubble from population (C) is indicated by red circles in Fig. 22 together with a very slow small bubble from the population (B), marked by green circles.

All these three types of bubbles oscillate in volume and collapse with the acoustic pressure field (compare the varying bubble sizes in Fig. 21), although their maximum expansions are not completely in phase with each other due to the different sizes. These types of bubble populations have been characterized before and are well-known [55], [41], [40], although they are not completely understood yet.

The inversion of motion (up instead of down) with growing bubble (equilibrium) size is apparently due to an increase in upward pointing primary Bjerknes force. Thus, the force due to liquid streaming, which is directed mainly downwards and carries the small bubbles, can be overcome once the bubble is beyond the Blake threshold and oscillating stronger. The jetting during collapse is induced by the fast translation [41] and/or by neighboring bubbles.

**Fig. 23:**
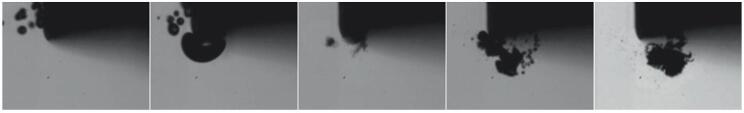
Images taken at the edge of the sonotrode tip (interframe time 199.8 μs, exposure time 1 μs, width 1.11 mm). Bubbles move down the side wall and then towards the center of the tip. Here a small cluster of bubbles is tracked that finally merges into a larger bubble.

With increasing the applied voltage to high amplitudes, the length of the bubble jet increases, but the behavior of large, medium and small bubbles in the structure remains similar. Moreover, in the high amplitude regime the (mostly medium-sized) bubbles that are near or at the lateral surface of the sonotrode move down next to the sonotrode wall. After reaching the edge they become larger and move towards the (A)-bubble in the middle of the sonotrode tip surface to merge with it. Fig. 23 illustrates the behavior of such bubbles.

### 5.3 Discussion

The proposed model is able to reproduce main features of the cavitating flows for the studied sonotrode setup while some aspects differ. Furthermore, the calculations give explanations to several observed phenomena.

The presence of the vertical jets is correctly predicted by the calculations. Their extents also match to the experimental results for different sonotrode displacement amplitudes. In the case of low amplitude the structure shown in Fig. 20 on the left hand side is approximately 5 mm long with medium sized bubbles moving towards the sonotrode. The corresponding calculation in Fig. 18a also demonstrates bubbles moving towards the sonotrode. Moreover, it shows that the pressure amplitude of the Blake threshold (for the considered bubble sizes in the range of 103 kPa to 112 kPa) is 5 mm beneath the sonotrode center coinciding with the jet extent. Consequently, the structure seen in the experiment is characterised by the inertially oscillating bubbles. For $d_{0}=5\;\mu m$, the bubbles are trapped within the region of the Bjerknes force reversion close to the sonotrode. In the case of $d_{0}=10\;\mu m$, the bubbles escape this area and populate the jet. The results from the experiment show that the real behavior exhibits features from both calculations. There is a confinement of bubbles close to the sonotrode tip and there is also a bubble jet extending into the domain. We suspect that one of the main reasons for this behavior in calculations is an insufficient margin of the considered sizes in the bubble population. The curves from Fig. 4 exhibit their probability maxima for the smallest radii. Obviously, these are not the global maxima since the probability for the smallest bubble sizes in a population should tend to zero. This might also explain the lack of the smaller-sized (B)-bubbles moving away from the sonotrode in the calculations. Other causes for deviations of the numerical results from the experimental ones are lack of the secondary Bjerknes force modeling and bubble injection only at the boundaries. Furthermore, a large oscillating bubble attached to the sonotrode cannot be reproduced by the model since the bubbles are assumed to be spherical and free oscillating. Finally, our analysis of the Eckart streaming [15] (not shown here) for the given frequency and acoustic power showed that this type of forcing accelerates the liquid by only several mm/s and has no impact on the overall flow behavior. Thus, it was not included in the present calculations.

The behavior of the bubbles moving from the lateral surface of the sonotrode to its bottom (Fig. 23), is reproduced by the model (Fig. 19). The bubbles move along the lateral wall with low velocities due to the flow induced by the jet. After reaching the edge of the sonotrode, the bubbles start to oscillate strongly and accelerate due to high pressure amplitudes. This is observed in both the calculations and the experiments.

## 6 Conclusion and outlook

In the present work, we developed a numerical solver able to calculate an acoustic field in a liquid and its effect on cavitation, flow and bubble transport. We utilized the Euler–Lagrange method, where the bubbles are depicted as single particles or a parcel of several identical bubbles. The Bjerknes force resulting from the interaction between the bubbles and the ultrasound field was implemented. To obtain the coupling terms from the Louisnard model, a single bubble equation including heat conduction (like Toegel’s model [51]) needs to be solved for each bubble for several oscillation periods. Such a procedure was not feasible during the simulation from the performance perspective for the targeted setups, since several millions or even billions of bubbles need to be represented. Therefore, a method based on interpolation tables was developed and implemented. In this method, Toegel’s model is solved as a pre-processing step given a suitable range of acoustic pressure amplitudes and of a bubble size distribution. The integral quantities needed by the damping and bubble motion models are calculated and tabulated. The tables are then used by the Euler–Lagrange solver.

We have verified our approach by considering a setup with a 12 cm sonotrode proposed in [29] and comparing the corresponding results. Since the parameters such as initial void fraction and bubble size distribution are not available from the experiments in most cases, we have performed two parameter studies to clarify what their influence is.

The bubble size distribution clearly affects the level of the acoustic pressure amplitude. The highest values of the acoustic pressure field correspond to the Blake threshold pressure of the largest bubbles from the population. Consequently, the polydisperse cases are similar in this regard when the same range of bubble sizes is considered. Additionally, the population type indirectly influences the velocity of the liquid, which originates from the momentum transferred from the bubbles. The primary Bjerknes force decreases severely, when the pressure drops below the Blake threshold. For the polydisperse cases this is the case for the most part of the population while in monodisperse cases all the bubbles are driven by the pressure amplitudes around the Blake threshold. Consequently, the polydisperse distributions show lower velocities than the monodisperse ones. As a conclusion, we recommend to include a realistic population in the calculations.

The second parameter study showed that the acoustic pressure amplitude is weakly dependent on the initial void fraction. On the contrary, the liquid velocity is strongly affected, e.g. it is twice as high in case of the 20-fold bubble fraction. Due to similar acoustic pressure the Bjerknes force does not differ strongly between the cases with low and high number of bubbles. Consequently, the liquid in the numerical setups with larger void fractions experiences higher momentum transfer from the bubbles and moves faster.

Furthermore, a validation was performed with a 1cm sonotrode setup. A phenomenon observed before [55], [39], [41], [40] and in the present experiments, where bubbles move against the flow, was explained by the calculations performed. The reason behind is the nature of the primary Bjerknes force whose direction is reversed when exceeding sufficiently large pressure amplitudes (Bjerknes threshold). We also observed bubbles moving along the lateral sonotrode walls to the sonotrode bottom in both the experiments and the numerical calculations. There were some discrepancies between the calculations and the experiments for higher sonotrode excitations. We suspect the main reasons for this to be an insufficient margin of the bubble population, no included models for the bubble interaction between each other and for the large bubble attached to the sonotrode surface. In the future, these should be addressed by including smaller bubble sizes in the population (up to 0.1μm), modeling the coalescence and breakage of the bubbles. The behavior of the large bubble should be studied with a scale resolving simulation (Euler-Euler calculation of a single bubble similar to [23]) and a model for the present Euler–Lagrange approach may be developed.

From the above we conclude that our model is able to depict important features of acoustically cavitating flows. One additional aspect which may be improved is the bubble seeding. The approach used in this work assumes that the bubbles are only seeded at solid boundaries, which is confirmed by the experiments but is not the only source of new bubbles. The motes present in any liquid, no matter how clean, serve as additional sources of bubbles when combined with gas dissolved in the liquid. An addition of the dissolved gas to the Eulerian phase as a property would be the first step. Then, the amount of the dissolved gas in a cell is decreased when new bubbles are introduced. If the rectified diffusion of bubbles is included in the Lagrangian frame, the amount of dissolved gas may also increase when the bubble dissolves.

## Declaration of Competing Interest

The authors declare that they have no known competing financial interests or personal relationships that could have appeared to influence the work reported in this paper.

## Footnotes

1 A solver from the COMSOL software package [11]. Note that all other calculations in this section were conducted with the custom solver [24] implemented in foam-extend [53].
